# Interdigitated and Wave-Shaped Electrode-Based Capacitance Sensor for Monitoring Antibiotic Effects

**DOI:** 10.3390/s20185237

**Published:** 2020-09-14

**Authors:** Jinsoo Park, Yonghyun Lee, Youjin Hwang, Sungbo Cho

**Affiliations:** 1 Department of Health Science and Technology, GAIHST, Gachon University, Incheon 21999, Korea; jspark88@gc.gachon.ac.kr (J.P.); yong9la@gc.gachon.ac.kr (Y.L.); 2 Department of Biomedical Engineering, Gachon University, 191 Hambakmoero, Yeonsu-gu, Incheon 21936, Korea; 3 Department of Electronic Engineering, Gachon University, 1342 Seongnamdaero, Seongnam-si, Gyeonggi-do 13120, Korea

**Keywords:** antibiotics, bacterial viability, capacitance sensor, impedance spectroscopy, label-free, real time

## Abstract

Label-free and real-time monitoring of the bacterial viability is essential for the accurate and sensitive characterization of the antibiotic effects. In the present study, we investigated the feasibility of the interdigitated and wave-shaped electrode (IWE) for monitoring the effect of tetracycline or kanamycin on *Staphylococcus aureus* (*S. aureus*) and methicillin-resistant *S.* *aureus* (MRSA). The electrical impedance spectra of the IWE immersed in the culture media for bacterial growth were characterized in a frequency range of 10 Hz to 1 kHz. The capacitance index (CI) (capacitance change relevant with the bacterial viability) was used to monitor the antibiotic effects on the *S*. *aureus* and MRSA in comparison to the traditional methods (disk diffusion test and optical density (OD) measurement). The experimental results showed that the percentage of change in CI (PCI) for the antibiotic effect on MRSA was increased by 51.58% and 57.83% in kanamycin and control, respectively. In contrast, the PCI value decreased by 0.25% for tetracycline, decreased by 52.63% and 37.66% in the cases of tetracycline and kanamycin-treated *S. aureus*, and increased 2.79% in the control, respectively. This study demonstrated the feasibility of the IWE-based capacitance sensor for the label-free and real-time monitoring of the antibiotic effects on *S. aureus* and MRSA.

## 1. Introduction

Not only new species of multi-drug-resistant bacteria but also super bacteria have been continuously observed due to mutations that confer resistance against the number of antibiotics developed for the prevention and therapy of pathogenic bacterial diseases [1,2]. *Staphylococcus aureus* (*S. aureus*) is one of the pathogenic bacteria, which promotes infections by producing virulence factors and causes bacteremia, endocarditis, skin and soft tissue infections, as well as hospital-acquired infections. Under a long-term antibiotic treatment, the bacteria could develop resistance for certain antibiotics [3,4]. The engineered *S. aureus* such as methicillin-resistant *S. aureus* (MRSA) are antibiotic-resistant strains and resistive to a diverse spectrum of antibiotics [5,6]. For developing an effective prophylactic vaccine against these pathogenic bacteria, various traditional methods and techniques such as cell viable count, polymerase chain reaction (PCR), and the disk diffusion test were carried out. However, these traditional methods are time consuming and require specific functionalized materials for target detection [7,8,9]. To overcome these disadvantages, high-throughput screening techniques, such as fluorescence-based microplate assay, imaging-based single-cell morphological analysis (SCMA), surface-enhanced Raman spectroscopy (SERS), functionalized PCR, or impedance microbiology has been introduced [10,11,12,13]. These techniques have the advantages of prompt accuracy and sensitive antibiotics analyses in real-time measurements.

For the accurate and sensitive characterization of the effect of antibiotics, electrical impedance microbiology has gained interest in the last few decades due to its real-time and non-destructive monitoring of the bacteria [14,15,16]. The method is performed with two paired electrodes that can be immersed in the culture medium containing the bacteria to detect the electrical conductivity changes in the media based on the bacterial growth or death [17]. The measured electrical parameters such as conductance, capacitance, or interfacial electrode impedance can be analyzed to quantify the bacteria metabolism as well as the effects of the antibiotics [18]. The electrical impedance measurement using miniaturized comb-structured interdigitated microelectrodes (IMEs) has been employed to characterize the electrical properties of microscopic samples [19,20,21].

Using the IME sensor, the sensitivity for the detection of micro-sized specimens was improved when compared with typical macro wires or disk electrodes due to a low ohmic drop, a fast-established steady state, rapid kinetics reactions, and an increased signal-to-noise ratio [22,23,24]. Yang et al. fabricated a capacitance-based IME, consisting of 25 paired electrodes with a width of 15 μm width and spacing for the real-time monitoring of viable *Salmonella* measurement in a low-frequency range [25]. Jo et al. reported an aptamer-functionalized capacitance sensor array for the real-time detection of bacterial growth and antibiotic susceptibility to gentamycin [26]. Settu et al. developed a gold IME-based impedance sensor to detect *Escherichia coli* (*E. coli*) in human urine samples for the diagnosis of urinary tract infection. Additionally, they optimized the measurement frequency to increase the detection sensitivity of the *E*. *coli* concentration during growth [27]. However, due to the presence of stronger electric fields at the edge of the rectangular fingers, a typical rectangular-shaped IME has a non-homogenous sensing area over the electrode. To increase the homogeneity of the sensitivity fields resulting in an improved reliability of the electrode sensor, the morphology of the electrode needs to be properly designed to avoid the edge effect of the electric field distribution [28,29]. To this end, we developed the interdigitated and wave-shaped electrode (IWE) that was devoid of any sharp edges, and it was used for the electrical impedance monitoring of the protein or thin-film analysis [30,31].

In this study, we investigated the feasibility of the IWE as a capacitance sensor for label-free and real-time monitoring of the dynamic bacterial growth and the antibiotic effects on bacterial viability. The electrical impedance was measured during the culture of *S*. *aureus* or MRSA in the presence of two different types of antibiotics (tetracycline or kanamycin at 30 μg/mL), and the results were compared with the traditional disk diffusion method and OD measurement. Based on the experimental results, we discussed the feasibility of the IWE as a capacitance sensor for the non-destructive and real-time monitoring of the antibiotics effect on the bacteria.

## 2. Materials and Methods

### 2.1. Fabrication of IWE Using a Microbial Culture Dish

**Figure 1:**
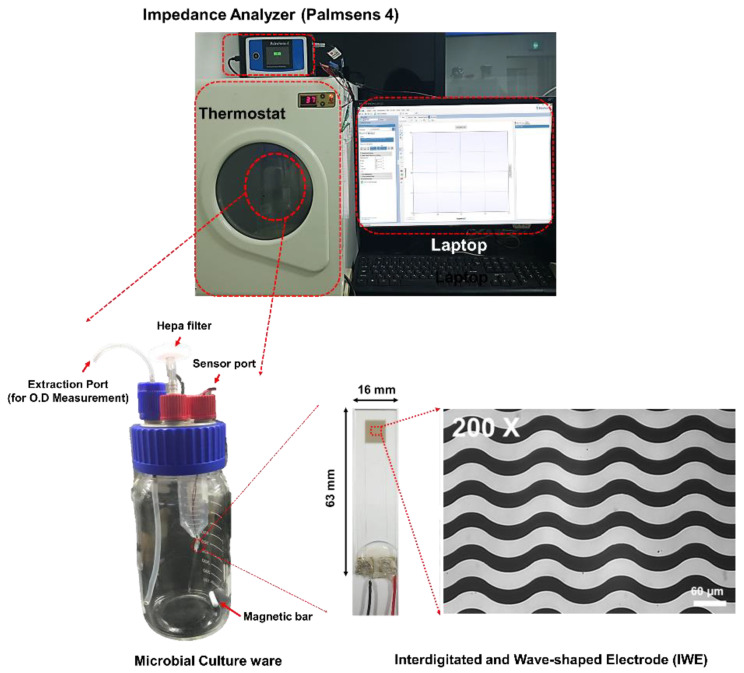
Schematic representation of the capacitance monitoring system for bacteria using the developed interdigitated and wave-shaped electrode (IWE) with microbial culture platform.

To detect the electrical impedance of the bacteria, we fabricated the IWE on a glass slide substrate (63 × 16 × 1.1 mm). The substrate was cleaned by ultra-sonication in 70% methanol. Following this, a negative photoresist (DNR L300-30, Dongjin Semichem co., Hwaseong, Korea) was spin-coated onto the substrate and patterned using photolithography to obtain a pattern with a spacing and width of 30 μm on IWE. Subsequently, a conductive layer was deposited on the patterned resist by electron beam evaporation (25 nm Ti and 50 nm Au), and the IWE was fabricated using the lift-off process. The IWE was placed in a modified microbial culture dish (DURAN GLS 80, Schott AG., Mainz, Germany) with an extract, and a sensor port was placed as shown in Figure 1. The terminal pads of the IWE were connected to a portable impedance analyzer (PalmSens4, Palmsens, Houten, The Netherlands).

### 2.2. Antimicrobial Susceptibility Testing

To check the susceptibility of bacteria to the antimicrobial agents, the Kirby–Bauer disc diffusion method was used according to the Clinical and Laboratory Standard Institute (CLSI) guideline of 2011 [32]. Each bacterial suspension was adjusted based on the McFarland standard value of 0.5 turbidities, swabbed onto Luria–Bertani (LB) broth, and incubated in the presence of antibiotic discs at 37 °C for 18 h. The antibiotics discs used were kanamycin (30 μg/mL) or tetracycline (30 μg/mL) (Liofilchem, Roseto degli Aburzzi, Italy), and the diameters of the inhibition zones were measured to determine the resistance or susceptibility of each isolate to these antibiotics.

### 2.3. Electrical Capacitance Monitoring

We inoculated 10 mL of MRSA or *S. aureus* culture of 0.6 optical density (OD) into 1 L of LB media in a cell culture flask along with a magnetic stirrer and incubated under constant stirring (250 rpm) at 37 °C in the thermostat (KRS200D, KARIS inc., Gyeonggi-do, Republic of Korea). During the 24 h incubation period, the capacitance spectra was measured in the frequency range of 10 Hz to 1 kHz for every 2-min time interval. The capacitance index (CI) or impedance magnitude index (ZI) termed for the change in capacitance or impedance was calculated using the following equation:

(1) $$\mathrm{CI}\;(\%)\;=\;\left|\frac{C_{0}-C_{\mathrm{mea}.}\;}{C_{0}}\right|\;\times \;100$$

(2) $$\mathrm{ZI}\;(\%)\;=\;\left|\frac{|Z{|}_{0}-{\left|Z\right|}_{\mathrm{mea}.}\;}{{\left|Z\right|}_{0}}\right|\;\times \;100$$

where C_o_ or |Z|_o_ and C_mea._ or |Z|_mea._ are the capacitance or impedance values measured at the initial time and after the cultivation period, respectively.

To compare the electrical capacitance results obtained using IWE sensor with that of the OD measurement, a sample was obtained using a sterile syringe, and its OD was measured using a spectrophotometer (Optizen POP, NEOGEN inc., Dae-jun, Republic of Korea) for every 2 h during the impedance measurement of 24 h. To check the effects of the antibiotics, 30 μg/mL of tetracycline or kanamycin was added into the culture when the OD of MRSA or *S. aureus* reached 0.6.

## 3. Results and Discussion

**Figure 2:**
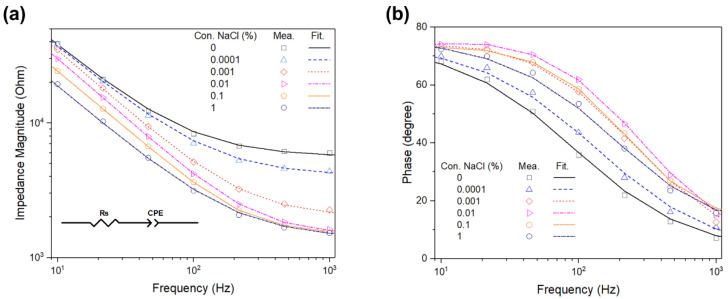
Electrical impedance spectra (**a**,**b**) of the IWE measured in different concentrations of NaCl electrolyte. The data points represent the measured (mea.) impedance spectra. The dotted lines represent the impedance spectra fitted (Fit.) using an equivalent circuit model, consisting of the solution resistance (R_s_), and a double-layer capacitance (CPE).

We evaluated the efficacy of the fabricated IWE for the real-time impedance monitoring of the bacteria by measuring the impedance spectra using different concentrations of NaCl as electrolyte in a frequency range from 10 Hz to 1 kHz, as shown in Figure 2. The electrical impedance characteristic of the electrode was governed by a sinusoidal signal with a frequency lower than 100 kHz applied to the electrodes; the bulk capacitance could be neglected, and the equivalent circuit can be simply designed using only an electrical conductivity of the bulk medium (R_s_) and the double-layer capacitance (CPE) in series [33,34].

Table 1 shows the data arrangement of the extrapolated values of the circuit parameters from the fitting results in Figure 2. As the concentration of NaCl was increased from 0 to 1%, the value of R_s_ decreased, but the increase or decrease in CPE depended on the NaCl concentration. The CPE is represented as T $(j\omega {)}^{p}$, where T and *p* are the adjusted parameters, and the results indicated the interfacial impedance of electrode, which can be attributed to the capacitive reactance characteristic [35].

Eventually, the designed equivalent circuit was well fitted to the measured impedance spectra of the electrolyte, and a fabricated IWE performance could be utilized for electrical impedance monitoring of the bacteria detection.

**Figure 3:**
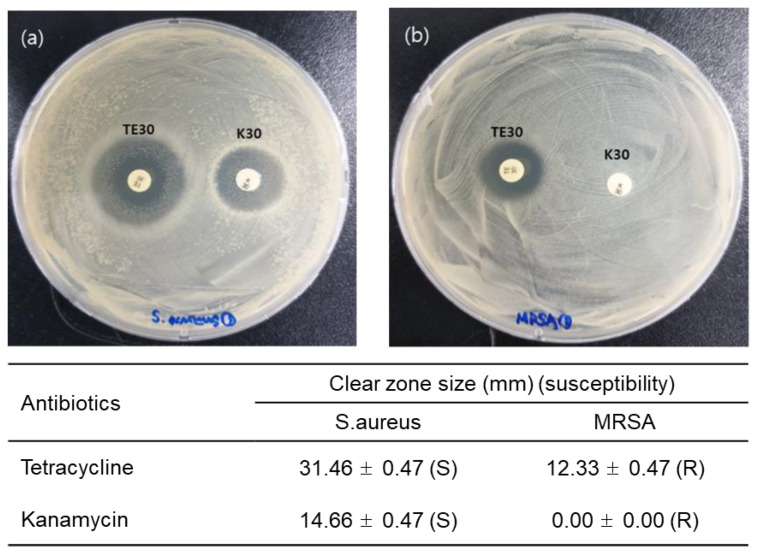
Antibiotic susceptibility test of (**a**) *S. aureus* or (**b**) MRSA was performed using discs containing the antibiotics, tetracycline 30 μg/mL (TE30), and kanamycin 30 μg/mL (K30), and by measuring the clear zone (S: susceptible, R: resistive).

For the analysis of the biological effect of antibiotics on MRSA or *S. aureus*, an antimicrobial susceptibility test was performed using the disc diffusion method based on the CLSI guidelines. The results show that when MRSA or *S. aureus* were treated with tetracycline and kanamycin at a concentration of 30 μg/mL, the clear zones of inhibition for *S*. *aureus* with diameters of 14.66 ± 0.47 mm and 31.46 ± 0.47 mm for kanamycin and tetracycline, respectively were recorded. On the other hand, a clear zone of inhibition for MRSA with a diameter of 10.33 ± 0.47 mm for tetracycline was found, while no effect was seen in case of kanamycin, as shown in Figure 3.

Based on the CLSI interpretative criteria, we confirmed that the MRSA was resistant to tetracycline (CLSI Criteria: *Staphylococcus* is resistant to tetracycline if the zone of inhibition is <14 mm diameter).

**Figure 4:**
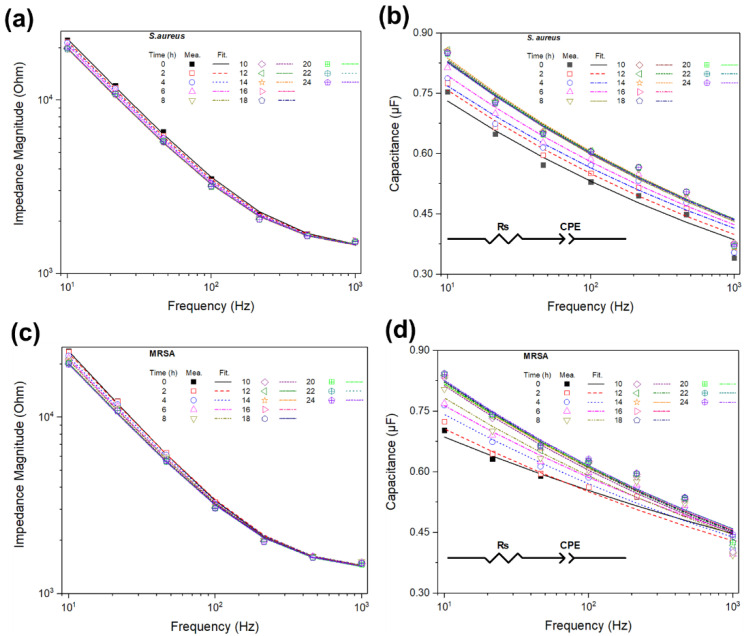
Impedance spectra and capacitance spectra for *S. aureus* (**a**,**b**) and methicillin-resistant *S. aureus* (MRSA) (**c**,**d**) during bacterial growth in Luria–Bertani (LB) medium. The data points represent the measured (mea.) impedance spectra. The dotted lines represent the fitting spectra (Fit.) using an equivalent circuit model, consisting of the solution resistance (R_s_) and a double-layer capacitance (CPE).

Figure 4 shows the raw data of the capacitance using the normalized capacitance spectrum of MRSA or *S. aureus* with fitting data using the equivalent circuit model as inserted in Figure 4. We have also shown significant changes in the rate of the normalized capacitance during the bacteria growth observed within the frequency range of 10 Hz to 1 kHz. Significant changes occurred at a frequency of 10 Hz for MRSA and *S. aureus*. As shown in Figure 4, the effect of the antibiotics on the bacterial growth curve was estimated using the OD at 595 nm and the normalized capacitance depending on the low-frequency range. Impedance-based detection methods have been used in microbiology to measure the conductivity of the medium using a paired interdigitated electrode submerged in the inoculated medium at a fixed frequency range of 10 Hz to 1 kHz [36,37].

Table 2 shows the extrapolated values of the equivalent circuit parameters from the fitting results in Figure 4. After the *S. aureus* or MRSA grew over a 24-hour incubation period, the value of CPE-T indicates that the capacitance increased, but the increase or decrease in R_s_ or CPE-*p* is dependent on the bacterial growth. Finally, the designed equivalent circuit was fitted to the measured impedance and capacitance spectra of the bacteria growth, and it was confirmed that the IWE performance could be utilized for electrical impedance monitoring of the antibiotic effects on the bacteria detection.

**Figure 5:**
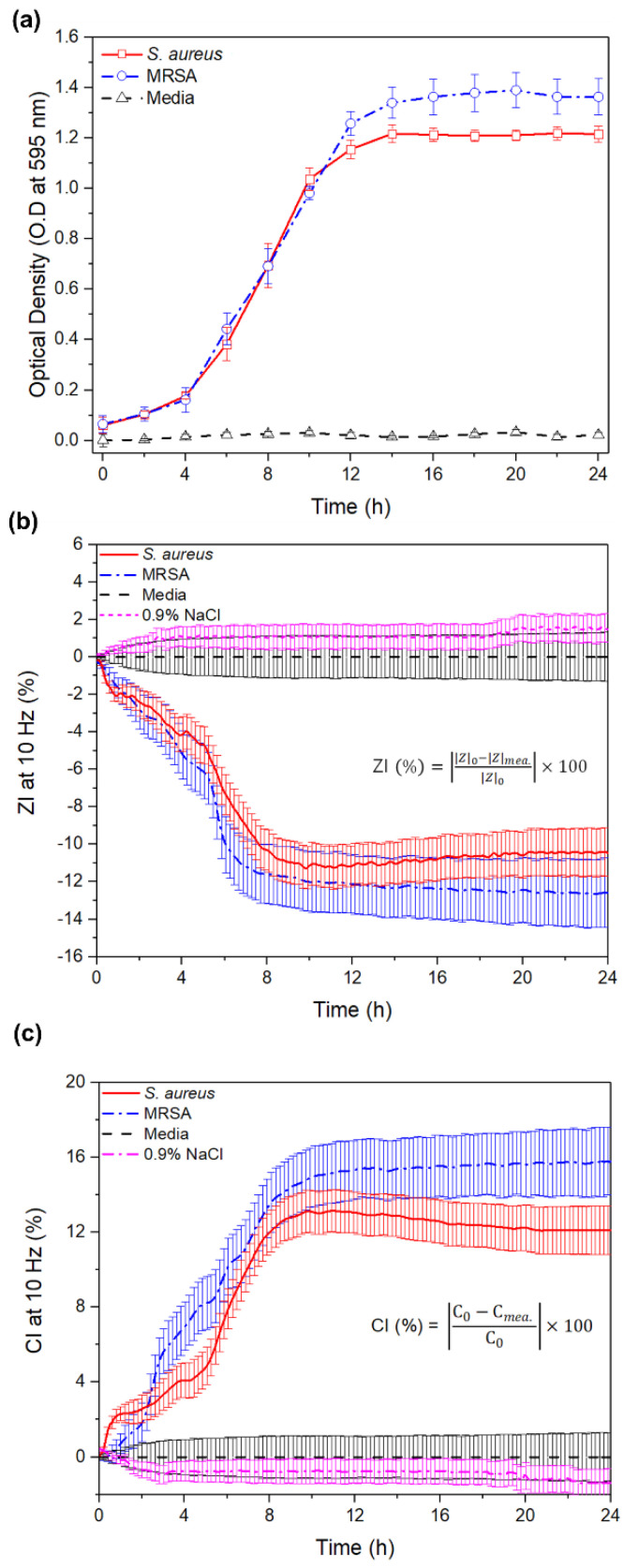
Typical bacterial growth curve measured over a period of 24 h at 595 nm (optical density (OD) = λ_595nm_) (**a**), and capacitance index (CI) and impedance magnitude index (ZI) for MRSA and *S. aureus* at 10 Hz (**b**,**c**) (standard error, *n* = 3).

From the comparison of the optical density measurement (OD at 595 nm) with the CI at 10 Hz for MRSA and *S. aureus* as shown in Figure 5a,b, the average of the value (*n* = 3) and standard error was measured during the culture. We observed a significant change in the CI at a fixed frequency of 10 Hz for MRSA or *S. aureus*, respectively. The CI at 10 Hz for MRSA was 13.49 ± 1.62 at 8 h of incubation, the corresponding OD value at the same time point was 0.68 ± 0.08, and for *S. aureus*, the CI at 10 Hz of 12.01 ± 1.12 and the corresponding OD value was 0.69 ± 0.07.

**Figure 6:**
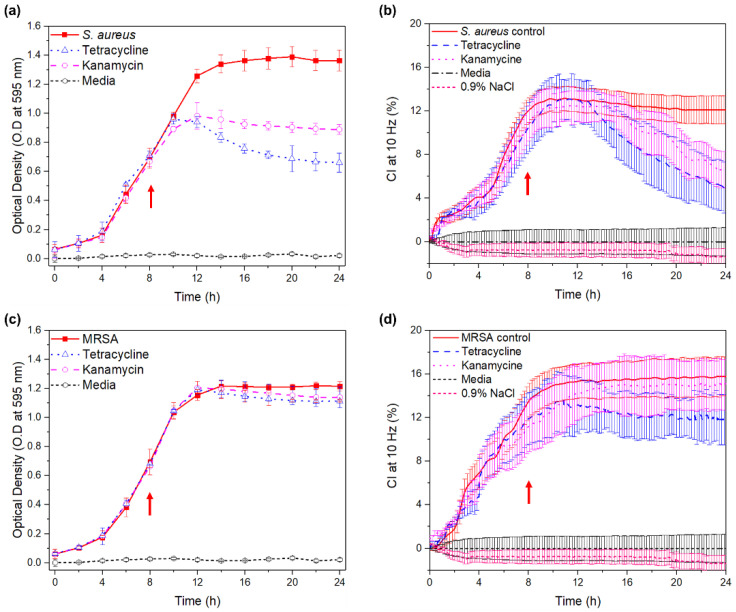
Effect of antibiotics (tetracycline or kanamycin, each of 30 μg/mL) on (**a**,**b**) *S. aureus* or (**c**,**d**) MRSA shown by the change in OD at 595 nm or CI at 10 Hz (arrow: antibiotics injection time).

Figure 6 depicts the quantified effect of antibiotics on MRSA and *S. aureus* using capacitive monitoring vs. OD measurement. The results indicated that when MRSA was treated with 30 μg/mL of tetracycline or kanamycin at 8 h, the CI at 10 Hz was increased from 9.51% to 15.01% and from 10.41% to 15.78% for kanamycin and control, and it was decreased from 11.88% to 11.85% for tetracycline, respectively. The PCI value was increased by 51.58% and 57.83% for kanamycin and control, while it was decreased by 0.25% for tetracycline, respectively. The measured OD value was increased by 63.6%, 70.1%, and 75.3% for tetracycline, kanamycin, and control, respectively. The PCI for the antibiotic effect on bacteria was calculated using the following equation:

(3) $$\mathrm{PCI}\;(\%)\;=\;\left|\frac{C_{8h}-C_{24h.}\;}{C_{8h}}\right|\;\times \;100$$

where *C*_8h_ and *C*_24h._ are the CI values measured at the antibiotic injection time point and then at the end of the cultivation period, respectively.

The CI at 10 Hz for the tetracycline or kanamycin-treated *S. aureus* was decreased from 10.43% to 4.94% at 10 Hz for tetracycline, decreased from 10.7% to 6.67% for kanamycin, and increased from 11.81% to 12.14% for the control. The PCI value decreased 52.63% for tetracycline-treated *S. aureus*, decreased 37.66% for kanamycin, and increased 2.79% for the control. The measured OD value decreased 31.25% for tetracycline, increased 1.12% for kanamycin, and increased 88.4% for the control, respectively.

Therefore, the effect of tetracycline or kanamycin on *S. aureus* was seen to vary depending on the effect of the antibiotics for a measurement period of 24 h. However, there were minimal changes in the OD or capacitive monitoring values for the MRSA treated with antibiotics for 24 h compared to the control group due to their resistance toward antibiotics. Assuming that the conductivity of medium changes due to the metabolism and growth of the bacteria in the media, the changes occurring in the CI is dependent on the bacterial growth under the effect of the antibiotics.

Based on these results, we demonstrated that the label-free and real-time bacteria impedance-monitoring platform made with the developed IWE placed in the microbial culture dish is feasible for the electrical capacitance-based monitoring of the microbial viability. The efficiency and the sensitivity of the IWE was demonstrated by evaluating the selective growth of either MRSA or *S. aureus* under the influence of the antibiotics depending on the frequency range. Therefore, when the developed IWE is placed in the microbial culture, it can be utilized as an impedance analyzer and can be potentially applied in pharmaceutical, cosmetics, agriculture, or food industries as a commercial product.

## 4. Conclusions

In this study, we investigated the feasibility of the IWE-based capacitance sensor for the label-free and real-time monitoring of the antibiotic effects on the bacterial viability. The electrical characteristics of the fabricated IWE were explained by fitting the designed equivalent circuit model to the measured impedance spectra of the sensor. The CI (change in capacitance value) of the IWE was derived for monitoring the bacteria growth and the antibiotic effects on the bacterial viability. The monitoring of CI using the IWE sensor was comparable with the experimental results of the typical disk diffusion test and the OD measurement. Thus, it was concluded that the IWE-based capacitance sensor is feasible for label-free and real-time monitoring of not only the bacterial growth but also the antibiotic effects on the bacterial viability.

## Author Contributions

J.P. performed the experiments for the electrical engineering part and wrote the manuscript. Y.L. performed the biological experiments. Y.H. conceptualized the experiments. S.C. provided support to procure all the experimental materials and supervised the work. All authors have read and agreed to the published version of the manuscript.

## Figures and Tables

**Table 1:** Extrapolated values of the designed equivalent circuit parameters from the fitting results in Figure 2.

Conc. NaCl	R_s_	CPE	
%	Ω	$T\;\left[\times 10^{-6}\;\Omega^{-1}\;s^{p}\right]$	*p*
0	4968.6 ± 107.7	0.747 ± 0.026	0.8639 ± 0.0012
0.0001	4128.3 ± 5.5	0.662 ± 0.001	0.8714 ± 0.0016
0.001	2101.3 ± 8.6	0.584 ± 0.003	0.9044 ± 0.0012
0.01	1465.1 ± 9.2	0.632 ± 0.008	0.9158 ± 0.0009
0.1	1401.0 ± 1.6	0.845 ± 0.006	0.9001 ± 0.0001
1	1363.2 ± 35.3	1.017 ± 0.005	0.8971 ± 0.0021

**Table 2:** Extrapolated values of the designed equivalent circuit parameters from the fitting results for *S. aureus* and MRSA in Figure 4.

	Time (h)	R_s_ (Ω)	CPE	
			$T\;\left[\times 10^{-6}\;\Omega^{-1}\;s^{p}\right]$	*p*
*S. aureus*	0	1327 ± 5.4	1.267 ± 0.021	0.861 ± 0.0005
	2	1330 ± 3.5	1.317 ± 0.022	0.860 ± 0.0012
	4	1338 ± 3.8	1.309 ± 0.045	0.865 ± 0.0024
	6	1340 ± 4.2	1.366 ± 0.032	0.863 ± 0.0018
	8	1335 ± 8.5	1.454 ± 0.018	0.858 ± 0.0032
	10	1336 ± 2.4	1.442 ± 0.022	0.860 ± 0.0011
	12	1340 ± 3.5	1.453 ± 0.015	0.859 ± 0.0008
	14	1330 ± 2.6	1.466 ± 0.011	0.858 ± 0.0004
	16	1346 ± 1.4	1.448 ± 0.019	0.859 ± 0.0015
	18	1335 ± 2.3	1.443 ± 0.022	0.859 ± 0.0021
	20	1348 ± 1.6	1.431 ± 0.032	0.861 ± 0.0032
	22	1341 ± 1.9	1.438 ± 0.021	0.861 ± 0.0024
	24	1341 ± 2.2	1.432 ± 0.018	0.861 ± 0.0018
MRSA	0	1343 ± 7.7	0.997 ± 0.026	0.907 ± 0.0012
	2	1323 ± 5.5	1.089 ± 0.014	0.891 ± 0.0021
	4	1341 ± 8.6	1.170 ± 0.035	0.885 ± 0.0015
	6	1330 ± 9.2	1.208 ± 0.022	0.885 ± 0.0005
	8	1335 ± 1.6	1.276 ± 0.015	0.877 ± 0.0007
	10	1326 ± 5.3	1.359 ± 0.064	0.870 ± 0.0011
	12	1312 ± 3.5	1.376 ± 0.032	0.870 ± 0.0010
	14	1318 ± 1.2	1.353 ± 0.015	0.873 ± 0.0021
	16	1313 ± 0.8	1.374 ± 0.054	0.871 ± 0.0014
	18	1314 ± 1.1	1.377 ± 0.023	0.870 ± 0.0011
	20	1310 ± 1.3	1.345 ± 0.011	0.874 ± 0.0015
	22	1311 ± 1.5	1.367 ± 0.025	0.872 ± 0.0019
	24	1314 ± 0.7	1.376 ± 0.032	0.871 ± 0.0013
